# Deriving Immune Modulating Drugs from Viruses—A New Class of Biologics

**DOI:** 10.3390/jcm9040972

**Published:** 2020-03-31

**Authors:** Jordan R. Yaron, Liqiang Zhang, Qiuyun Guo, Michelle Burgin, Lauren N. Schutz, Enkidia Awo, Lyn Wise, Kurt L. Krause, Cristhian J. Ildefonso, Jacek M. Kwiecien, Michael Juby, Masmudur M. Rahman, Hao Chen, Richard W. Moyer, Antonio Alcami, Grant McFadden, Alexandra R. Lucas

**Affiliations:** 1 Center for Personalized Diagnostics, Biodesign Institute, Arizona State University, Tempe, AZ 85281, USA; jyaron@asu.edu (J.R.Y.); liqiang.zhang@asu.edu (L.Z.); qguo27@asu.edu (Q.G.); mburgin@asu.edu (M.B.); lschutz2@asu.edu (L.N.S.); eawo1@asu.edu (E.A.); mjuby82@midwestern.edu (M.J.); 2 Center for Immunotherapy, Vaccines and Virotherapy, Biodesign Institute, Arizona State University, Tempe, AZ 85281, USAgrantmcf@asu.edu (G.M.); 3 Department of Oncology, Tongji Hospital, Tongji Medical College, Huazhong University of Science and Technology, Wuhan 430030, China; 4 University of Otago, Dunedin 9054, New Zealand; lyn.wise@otago.ac.nz (L.W.); kurt.krause@otago.ac.nz (K.L.K.); 5 Department of Ophthalmology, University of Florida, Gainesville, FL 32610, USA; ildefons@ufl.edu; 6 Department of Pathology and Molecular Medicine, McMaster University, Hamilton, ON L8S4L8, Canada; 7 The Department of Tumor Surgery, Second Hospital of Lanzhou University, Lanzhou 730030, China; chenhao3996913@163.com; 8 Department of Molecular Genetics and Microbiology, University of Florida, Gainesville, FL 32610, USA; rmoyer@mgm.ufl.edu; 9 Centro de Biología Molecular Severo Ochoa (Consejo Superior de Investigaciones Científicas and Universidad Autónoma de Madrid), Cantoblanco, 28049 Madrid, Spain; aalcami@cbm.csic.es; 10 St Joseph Hospital, Dignity Health, Creighton University, Phoenix, AZ 85013, USA

**Keywords:** virus, immune modulation, protein, serpin, chemokine binding protein, chemokine, growth factor, cytokine, interleukin, therapeutic, biologic

## Abstract

Viruses are widely used as a platform for the production of therapeutics. Vaccines containing live, dead and components of viruses, gene therapy vectors and oncolytic viruses are key examples of clinically-approved therapeutic uses for viruses. Despite this, the use of virus-derived proteins as natural sources for immune modulators remains in the early stages of development. Viruses have evolved complex, highly effective approaches for immune evasion. Originally developed for protection against host immune responses, viral immune-modulating proteins are extraordinarily potent, often functioning at picomolar concentrations. These complex viral intracellular parasites have “performed the R&D”, developing highly effective immune evasive strategies over millions of years. These proteins provide a new and natural source for immune-modulating therapeutics, similar in many ways to penicillin being developed from mold or streptokinase from bacteria. Virus-derived serine proteinase inhibitors (serpins), chemokine modulating proteins, complement control, inflammasome inhibition, growth factors (e.g., viral vascular endothelial growth factor) and cytokine mimics (e.g., viral interleukin 10) and/or inhibitors (e.g., tumor necrosis factor) have now been identified that target central immunological response pathways. We review here current development of virus-derived immune-modulating biologics with efficacy demonstrated in pre-clinical or clinical studies, focusing on pox and herpesviruses-derived immune-modulating therapeutics.

## 1. Introduction

**Figure 1:**
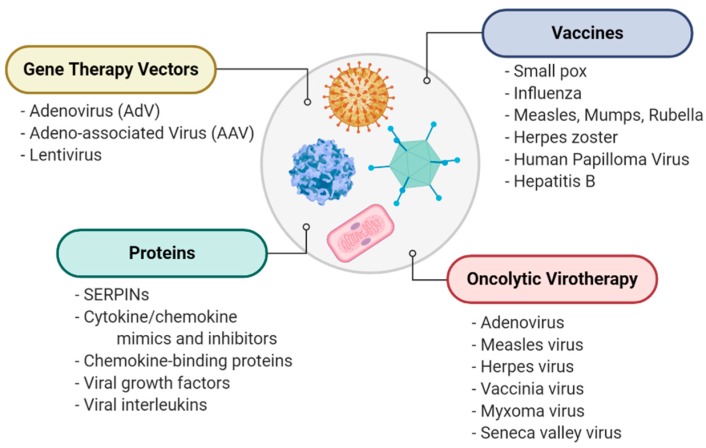
A general overview of therapeutic uses of viruses, including direct use as immunogens for vaccines, gene therapy vectors for delivery of therapeutic protein-coding sequences, tools for destroying cancer cells in oncolytic virotherapy and as sources for novel therapeutic proteins.

Historically, viruses were first investigated as a source of vaccines for the prevention of dangerous viral infections. In a key example, Variola caused billions of severe and deadly infections with widespread and lethal outbreaks in the past, albeit now eradicated worldwide in 1980 through World Health Organization vaccination programs. It was Jenner’s observation that milkmaids exposed to “Cowpox virus” were naturally protected against smallpox [1]. This was the basis for developing the first smallpox vaccine in 1796 leading to the current day attenuated vaccine strain, named vaccinia virus. Since that time effective vaccines for many infectious diseases, such as influenza, measles, mumps and rubella have been developed [2], continuing to save uncounted numbers of lives (Figure 1). More recently, the development of the Herpes zoster vaccine has reduced the incidence of painful shingles outbreaks and a human papillomaviral (HPV) vaccine is now given to young women (and more recently men as well) to prevent cervical cancer and other HPV-associated malignancies [3]. Further, hepatitis vaccines reduce the risk of hepatitis, liver damage and cirrhosis and offer the additional benefit of reducing the attendant risk for later hepatocellular cancer [4]. Vaccines thus represent the first therapeutic use of viruses as immune-modulating agents.

Whole viruses are also under investigation as new approaches to treating cancer, a field referred to as viral oncolytics, in which viruses selectively target and kill cancer cells as well as enhancing natural immune cell killing of cancer cells [5,6]. Adenoviruses, herpesviruses, measles, senecavirus and poxviruses, such as vaccinia and myxoma, are all in active development as treatments for many cancers, including multiple myeloma, melanoma, sarcomas and brain cancers such as glioblastoma multiforma (GBM) [7,8,9,10,11]. The rabbit-derived myxomavirus is one such virus now demonstrated to selectively target, infect and kill cancer cells, without infecting or killing normal, noncancerous mammalian and human cells [6]. Aside from acting as lytic agents, some viruses have been genetically modified, armed with therapeutic transgenes to activate the native T-cell-mediated immune responses and thus to suppress or kill cancer cells and prevent metastasis [12]. 

Viruses are also used as vectors designed to express genes that target genetic disorders or treat pathogenic processes. Viral vectors have been designed to express genes that replace defective gene expression in patients with congenital disorders including alpha-1-antitrypsin (SERPINA1; A1AT) deficiency, a genetic serpin disorder, or serpinopathy, which causes severe emphysema [13]. Adenoviral (AdV) and Adeno-associated viral (AAV) vectors provide systems for treating rare genetic disorders. One such vector, AAV8, has been reported to improve development in children with X-linked myotubular myopathy [14]. AAV expression of immune- and angiogenesis-modulating proteins are also in development for treating uveitis and macular degeneration [15,16]. Some AAV vectors have also recently been designed to ectopically express virus-derived immune-modulating proteins, the focus for this review [17]. 

In addition to the exploitation of viruses as vaccines, oncolytics and gene therapy vectors, viruses also provide an extensive repository in nature for new immune-modulating drugs [18,19]. The same large DNA viruses that cause severe disease such as poxviruses and herpesviruses have developed highly effective and potent methods to evade the immune responses of the host [20]. These virus-derived immune-modulating tools have evolved over millions of years in a virus versus cell arms race as counterattacks to host immune responses, effectively blocking immune responses by targeting central immune pathways [20]. These proteins are now under investigation for the development of immune-modulating biologics designed to treat severe immune diseases where the immune response has become excessive and/or dysregulated [13,14,15,16,17]. Poxviruses ranging from vaccinia to myxoma and variola, as well as herpesviruses, ranging from mouse gamma herpesvirus to cytomegalovirus, all encode extraordinarily effective immune-modulating proteins that bind and inhibit proteases, cytokines and chemokines, while yet other proteins are homologs or decoys for cytokines, cytokine receptors and growth factors. 

Patients that may benefit from new therapeutics which address limitations of existing treatments include those with autoimmune and inflammation-based diseases such as rheumatoid arthritis, psoriasis, psoriatic arthritis, inflammatory bowel disease (Crohn’s), inflammatory vascular diseases, stroke, myocardial infarction, unstable angina, heart failure, sepsis, diabetes, renal failure and central nervous system trauma [21,22,23,24,25]. Other targets could include allograft transplant rejection, which is associated with elevated inflammatory serine proteases, chemokines and cytokines together with complement activation in the circulating blood and affected organs [26]. Increases in tumor necrosis factor (TNF), interferon-gamma (IFNγ), interleukins (IL-1β, IL-18 among others), chemokines, growth factors and complement components are frequently used as biomarkers to track the progression of disease in these immune system-driven conditions and many of these same immune system components are targeted, blocked or redirected by viruses [20]. Virus-derived immune-modulating proteins thus provide a natural source for treatments designed to control dysregulated responses in both the innate (inflammatory) and acquired (antibody- and cell-mediated) arms of the immune system, where aggressive damage to the vasculature and end organs clinically progress to a wide spectrum of inflammatory and autoimmune disorders. 

**Figure 2:**
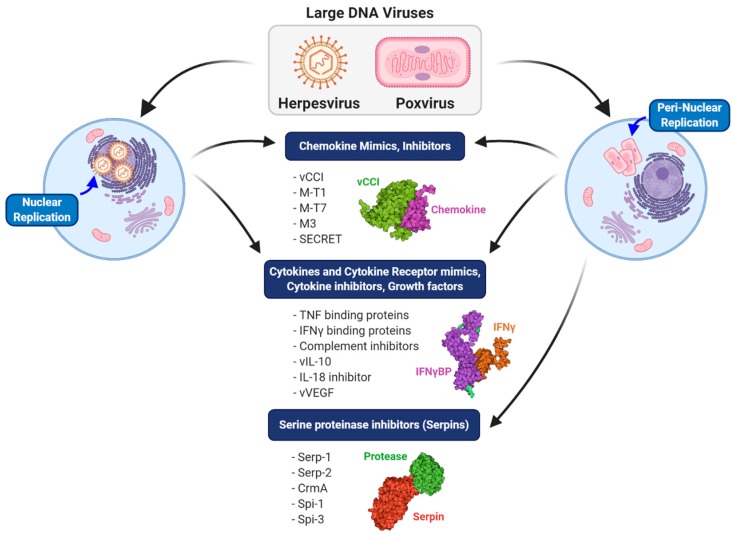
Comparison of different classes of immune modulators produced by large DNA viruses. Herpesviruses, which replicate in the nucleus, and poxviruses, which replicate in a peri-nuclear factory, both produce chemokine mimics and inhibitors, cytokine and cytokine receptor mimics and inhibitors and growth factor mimics. A major distinguishing feature of poxviruses is the production of both intracellular and extracellular serine protease inhibitors (serpins).

Immune-modulating protein biologics and antibodies are actively being identified and developed as new treatment options for immune disorders. The virus-derived biologics that have been tested for potential therapeutic uses range from cytokine and chemokine mimics to receptor mimics, growth factors and regulators of the coagulation cascade and complement systems, all representing naturally derived proteins evolved to provide extraordinarily effective agents. Interestingly, many of the immune modulators identified at present have been developed from herpesviruses or poxviruses, owing to the comparatively large sequence space in their DNA genomes for adopting and evolving new immune-modulating virulence factors (Figure 2). The majority of studies involving virus-derived protein biologics have been pre-clinical (i.e., laboratory animal models), but one virus-derived serpin has been tested in a Phase IIa trial in patients with unstable coronary syndromes, proving safe and effective [27], thus paving the way to future trials of this new class of virus-derived protein biologics. With this review, we focus on studies where virus-derived biologics have been investigated as immune-modulating treatments. We begin with an overview of serpins, one of which has been tested clinically, followed by a review of chemokine modulating proteins and finally virus-derived mimics and inhibitors of cytokines and growth factors and ending with an overview of the discovery of unique modulators derived from other viruses and organisms that have demonstrated efficacy in preclinical studies. These virus-derived biologics represent a new and almost untouched source for highly potent immune-modulating therapeutics.

## 2. Serine Protease Inhibitors (Serpins)

**Figure 3:**
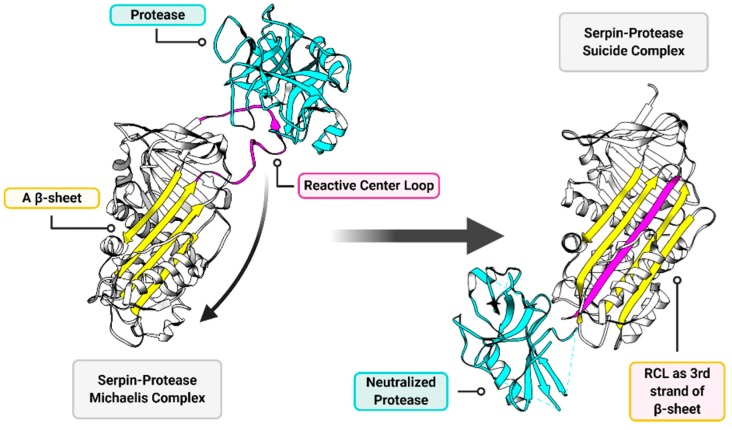
General overview of serpin function. The two major functional components of a serpin structure are the reactive center loop (RCL; magenta) and the A β-sheet (yellow). The RCL presents a protease cleavage site as a substrate to serine (and cysteine in cross-class serpins) proteases (cyan). When the target protease acts upon the RCL substrate a transient Michaelis complex forms (left), wherein the serpin and the protease are temporarily covalently bound to each other. When this Michaelis complex forms, the RCL performs a dramatic rearrangement and inserts as the third of five strands in the A β-sheet (right). The deformed and neutralized protease and serpin are permanently linked in a suicide complex, which then gets degraded. Structures are modeled on RSCB entries 1K9O (left) [36] and 1EZX (right) [35].

Serpins are ubiquitous serine protease inhibitors that function as regulatory across all kingdoms of the Tree of Life, found in diverse organisms ranging from plants and fish to insects and humans [28]. Likely due to horizontal gene transfer, some viruses also express serpins [28]. In mammals, serpins regulate pathways central to thrombosis (coagulation), thrombolysis, complement activation, apoptosis, connective tissue degradation, immune cell activation and even neuronal and endocrine pathways [28,29,30,31,32]. Serpins function as suicide inhibitors providing an external strained reactive center loop (RCL) that presents a P1-P1′ scissile bond as bait for cleavage by targeted proteases [31]. The proteases that are targeted by serpins then cleave the RCL at the P1-P1′ bond, becoming covalently bound to the cleaved end of the RCL in a Michaelis complex, which is normally transient in an enzyme–substrate complex [33,34]. The cleaved RCL with the bound protease then folds in a dramatic rearrangement into the A β-sheet (as the third of five strands), bringing the now-deformed and neutralized protease a distance of more than 70Å to the opposite pole of the serpin in a stable, inactive serpin-enzyme complex [35] (Figure 3).

Serpins share approximately 30% homology across multiple kingdoms, including plants, mammals and bacteria, in addition to viruses. The impact of serpins on the regulation of multiple central pathways is evident in the prevalence of serpins in the circulating blood, representing 2%–10% of circulating plasma proteins [37]. Further, they are expressed after injury in many tissues at high levels [38]. Due to their suicide inhibition mechanism, forming 1:1 inhibitory complexes, serpins are understood to require high available concentrations for the effective blockade of protease activation [31]. The impact of serpin-mediated function is also seen in genetic disorders such as alpha-1-antitrypsin (SERPINA1; A1AT) deficiency causing severe emphysema [39], genetic mutations in plasminogen activator inhibitor-1 (SERPINE1; PAI-1) and antithrombin III (SERPINC1; ATIII) [40] leading to clotting disorders, genetic deficiency of the serpin complement C1 inhibitor (SERPING1; C1INH) causing angioedema [41], or neuroserpin mutations (SERPINI1; NSP) causing epilepsy and other neurological disorders [42,43]. Conformational problems with serpins, usually caused by mutations, are termed *serpinopathies*, wherein chains or aggregates of inactive serpins are postulated to form inactive polymers where the mutated serpin RCL is inserted into the beta-sheet of adjacent serpins or via a domain-swapping mechanism with a similar outcome [44]. Protein replacement therapy, as well as AAV expression of A1AT expression, are in development to treat A1AT deficiency, the archetypal serpinopathy [39].

The impact of serpins on normal regulatory functions in mammals is evident by the evolution of poxviruses to express immune-modulating serpins that block host immune defenses against viral infections. When expressed, select viral serpins markedly increase virulence with profound effects on the ability of the infected host to mount an immune response against the infecting virus. In Myxomavirus infections, the deletion of Serp-1 or Serp-2 genes modifies this highly lethal infection in European rabbits (*Oryctolagus cuniculus*) to a benign local infection [45,46]. The Shope fibroma Myxomavirus variant has a scrambled Serp-1 gene sequence, and produces only a benign self-limiting infection in rabbits [45,46,47]. Poxviral serpins act as highly effective inhibitors, functioning at picomolar concentrations [27,48,49,50,51]. Serpins thus have extensive and highly evolved immune modifying and suppressive functions allowing poxviruses to infect and spread within the host. 

Based on this exceptional activity the potential for several virus-derived serpins to function as immune-modulating biologics has been investigated. The orthopoxviruses express three serpins, Spi-1, Spi-2 (CrmA) and Spi-3 while the leporipoxvirus, Myxomavirus, expresses three serpins, Serp-1, Serp-2 and Serp-3. The poxvirus-derived serpins and serpin RCL-derived peptides are discussed in the following sections, describing molecular targets and findings in current preclinical and clinical studies, demonstrating for some serpins the potential for therapeutic benefit (Table 1).

### 2.1. Serp-1

Serp-1 is a 50–55 kDa glycosylated Myxomavirus serpin that inhibits both thrombotic (factor X and thrombin) and thrombolytic (tissue- and urokinase-type plasminogen activators {tPA and uPA} and plasmin) serine proteases [67,68]. Serp-1 has been tested in perhaps the broadest array of preclinical models amongst all virus-derived immune modulators that have been tested as potential therapeutics. Serp-1 was first tested in a rabbit model of aortic balloon angioplasty injury and later after balloon angioplasty and stent implant, demonstrating significantly reduced inflammation and plaque growth [53]. Serp-1 efficacy was detected at picogram-to-microgram doses after both single and multiple systemic injections. Subsequently, effective inhibition of arterial inflammation and plaque growth was seen in aortic [48], renal [54] and heterotopic heart transplants [55] in mice and rats. More recently, Serp-1 has proven effective in severe inflammatory vasculitic syndrome models in mice after MHV-68 herpes virus infection in IFNγR deficient mice [56] and, in a different study, after aortic xenograft implants of human temporal artery biopsy specimens from patients with suspected inflammatory vasculitic syndromes (Giant cell arteritis) into the abdominal aorta of severe combined immunodeficient (SCID) mice [57]. In other work, Serp-1 treatment reduced inflammation in collagen-induced arthritis in rats [58] and reduced plaque in a mouse carotid compression model [59]. When delivered locally by subdural infusion Serp-1 reduced inflammation and improved functional recovery in a balloon crush spinal cord injury model in rats [60]. These prior studies were performed by systemic injection of Serp-1 either by intraperitoneal or intravenous injection or by subdural infusion to the spinal cord in rats. When delivered topically in saline solution or via a chitosan-collagen biocompatible hydrogel sustained-release substrate, Serp-1 accelerated healing in a full-thickness wound healing model in C57BL6/J mice, with improved vascularization and tissue remodeling [52]. These diverse studies attest to the marked capacity and the potential of this protein to function as a biologic therapeutic. 

Mechanistically, multiple studies point to a critical role of the urokinase-type plasminogen activator receptor (uPAR) in the function of Serp-1 in vivo. In immunoprecipitation studies, Serp-1 binds to uPAR and co-precipitates with the actin-binding and coordinating protein filamin B [69]. Serp-1 efficacy is abrogated in a uPAR-deficient mouse aortic allograft models [70]. Similarly, Serp-1-mediated promotion of full-thickness wound healing is completely blocked by co-treatment with a neutralizing antibody to uPAR [52].

Of central interest to the translational potential of virus-derived immune-modulating biologics, Serp-1 was examined in patients with unstable coronary syndromes, unstable angina and small heart attacks with coronary stent implant, in the first human clinical trial of a viral protein. Serp-1 was tested in randomized, blinded FDA-approved Phase 1 (safety) and 2 (Safety and efficacy) clinical trials using a good manufacturing product (GMP) protein expressed in Chinese hamster ovary cells (CHO) cells [27]. In a clinical trial, Serp-1 proved safe with minimal, if any, side effects and no major adverse cardiovascular events (MACE score = 0), and specifically with no detected increase in thrombosis or bleeding and no increased infections. In the Phase 2 trial (NCT00243308), Serp-1 treatment was tested in a randomized, blinded, dose-escalating trial in patients with documented unstable coronary syndromes (patients with Non-ST elevation MI and unstable angina who required coronary angiography and had demonstrated indications for coronary stent implant). In this trial, performed at seven sites in Canada and the United States, no side effects were reported with a reported MACE of 0 at the highest dose (15 µg/kg IV × 3 days). Further, no neutralizing antibodies were detected after Serp-1 infusions. Serp-1 treatment significantly reduced markers of myocardial damage, Troponin I and creatinine kinase, TNI and CK respectively. No reduction in plaque size was detected by intravascular ultrasound but the total trial included only 48 patients in total. Thus, this clinical trial, which was a *first-in-class* clinical trial of a virus-derived biologic in man, proved treatment with a viral serpin safe and effective in reducing markers of cardiac damage and proved safe with no significant antibody production. In summary, while this clinical trial did not detect reduced plaque growth after coronary stent implants, perhaps due to the small patient cohort size, Serp-1 treatment given for three days after stent implant did significantly reduce markers of heart damage at the highest dose, a predictor of longer-term outcomes in ischemic heart disease.

### 2.2. Serp-2

Serp-2 is a 34 kDa serine and cysteine (cross-class) protease inhibitor, derived from Myxomavirus that inhibits both apoptotic (granzyme B, a serine protease, and caspase-8 and -10, cysteine proteases) and inflammasome (caspase-1, a cysteine protease) pathways [71,72,73]. In mouse models of aortic allograft transplants, Serp-2 significantly reduced inflammation and intimal hyperplasia, again with no detected side effects [50,51]. In a model of partial 70% warm ischemia-reperfusion injury in the liver (LIRI), Serp-2 treatment given systemically also improved survival over 10 days, reduced necrotic damage of the liver and lowered acute markers of liver damage [61]. Surprisingly, caspase-1, caspase-3 and caspase-8 activation were not suppressed, suggesting an alternative mechanism of protection potentially by inhibition of circulating inflammatory proteases. When tested in a mouse carotid cuff compression model of atherosclerosis, Serp-2 treatment had a demonstrated trend toward the reduced carotid plaque, but significantly reduced proximal aortic root plaque growth as a systemic effect on vasculature proximal to the carotid injury [59]. This systemic efficacy of Serp-2 is not reproduced by the infusion of an inactive Serp-2 RCL mutant nor, surprisingly, by the Cowpox analog CrmA that has similar molecular targets to Serp-2 (see next section). When Serp-2 is given to mice after implant of granzyme B-deficient aortic transplants, the efficacy for reducing graft vasculopathy is lost, indicating that Serp-2 immune-modulating functions in this transplant model are at least in part dependent upon blockade of granzyme B and apoptosis [50].

### 2.3. CrmA

CrmA (cytokine response modifier A) is a cross-class serpin expressed by Cowpox virus, with analogs in other orthopoxviruses such as vaccinia virus or ectromelia virus referred to as SPI-2 [74]. CrmA binds granzyme B and caspases 1 and 8 with higher affinity than Serp-2 [73]. Despite the higher affinity, when CrmA and Serp-2 genes are interchanged in viruses they did not restore the immune-modulating properties of the alternate gene, nor did they increase virulence [71]. As mentioned above, in a mouse aortic transplant model, Serp-2 but not CrmA reduced aortic allograft inflammation and intimal hyperplasia, indicating a difference in potential for therapeutic efficacy [50]. However, some preclinical models have shown efficacy for CrmA as a therapeutic approach. Pre-treatment with an adenovirus delivering the coding sequence for CrmA potently inhibited anti-Fas antibody-induced fulminant hepatitis in male BALB/c mice [62]. In this model, protection by CrmA was adenovirus dose-dependent and associated with the dramatic reduction in TUNEL staining, caspase-3 activation and CD11b-positive cell infiltration. In similar work, adenoviral transduction of CrmA protected mice from concanavalin-A-induced hepatitis, with an associated reduction in TUNEL staining, caspase-3 activation, CD11b-positive cell infiltration and IL-18 secretion [63]. Interestingly, CrmA did not affect T-cell phenotypes in this model, despite concanavalin-A hepatitis classically being thought of as driven by T-cells [75]. In a doxorubicin-induced model of cardiomyopathy in mice, cardiac-specific expression of CrmA significantly improved early survival, but the effect was transient and by twelve days post-induction no benefit could be observed [65]. In this study, CrmA expression stably suppressed activity of caspase-3, caspase-8 and caspase-9, but was unable to reduce levels of TUNEL staining or the release of apoptosis-inducing factor (AIF). These results indicated that while CrmA did successfully prevent initial caspase-3/8/9-dependent apoptosis, redundant pathways were engaged ultimately resulting in cardiac damage by an apoptotic caspase-independent mechanism. Like Serp-1 and Serp-2, CrmA has also been tested as a recombinant protein therapeutic. CrmA conjugated to an HIV trans-activator of transcription (TAT) domain, which confers cell permeability, potently reduced disease severity and improved survival in both anti-Fas-induced fulminant hepatitis, doxorubicin-induced heart failure and left anterior descending (LAD) artery ligation-induced myocardial infarction in mice [64].

### 2.4. Serpin-Derived Peptides

In initial studies with the Myxomavirus derived serpin, single or limited doses of Serp-1 given immediately after vascular injury or transplant produced a prolonged activity detected in preclinical animal models of vascular disease. As the RCL for serpins is cleaved, it was postulated that naturally derived metabolites from the Serp-1 RCL might have an activity that can extend Serp-1 activity. The *in vivo* circulating half-life of Serp-1 is reported as short and dependent on the organism, with measurements of 3 min in mice [59], 20 min in humans [27] and up to 12 or more hours in rabbits [76]. However, much of the activity of Serp-1 may be due to non-circulating or tissue-based activity and or active metabolites. Thus, 1 to 10 days of Serp-1 treatment given systemically has had up to 30 days benefit, and for the inflammatory vasculitic syndrome (IVS) model of herpesvirus-induced vasculitis, 10 to 30 days of Serp-1 treatment led to improved survival up to 150 days [56]. A series of Serp-1 and mammalian neuroserpin (NSP) RCL peptides were thus tested; peptide sequences based upon natural cleavage sites predicted by the Expasy/PeptideCutter program [33,66]. One of the Serp-1 RCL peptides, designated S-7, reduced inflammation after aortic allograft transplant in mice, as well as improved survival with similar efficacy to Serp-1 in the MHV-68 IVS mouse model in IFNγR^−/−^ mice. In contrast, Neuroserpin RCL peptides were not active in the aortic transplant model. Thus, bioinformatically predicted RCL peptides derived from Serp-1 have demonstrated preclinical efficacy in IVS and transplant vasculopathy models unique to the virus-derived parent protein. 

Serp-1 and the Serp-1 S-7 peptide lose therapeutic efficacy and no longer improve survival when the gut bacterial microbiome is suppressed with broad-spectrum antibiotics [33]. This finding suggests that this Myxomaviral immune regulating serpin has tapped into trans-kingdom interactions and host defenses that modify host immune responses between the infecting virus and the host bacterial microbiome. These peptides, while expected to inhibit serine proteases, instead bind and inhibit mammalian serpins such and PAI-1 and NSP [67]. When the Serp-1 RCL peptide S-7 was modified to improve hydrogen bonding in the A β-sheet, the resultant peptides MPS7-8 and MPS7-9 displayed extended activity improving survival, even in antibiotic-treated mice in the IFNγR^−/−^ [33]. Thus, immune-modulating biologics may be sensitive to host environmental factors and may require molecular optimization to maintain therapeutic efficacy. 

## 3. Chemokine Binding Proteins (CBPs) 

**Figure 4:**
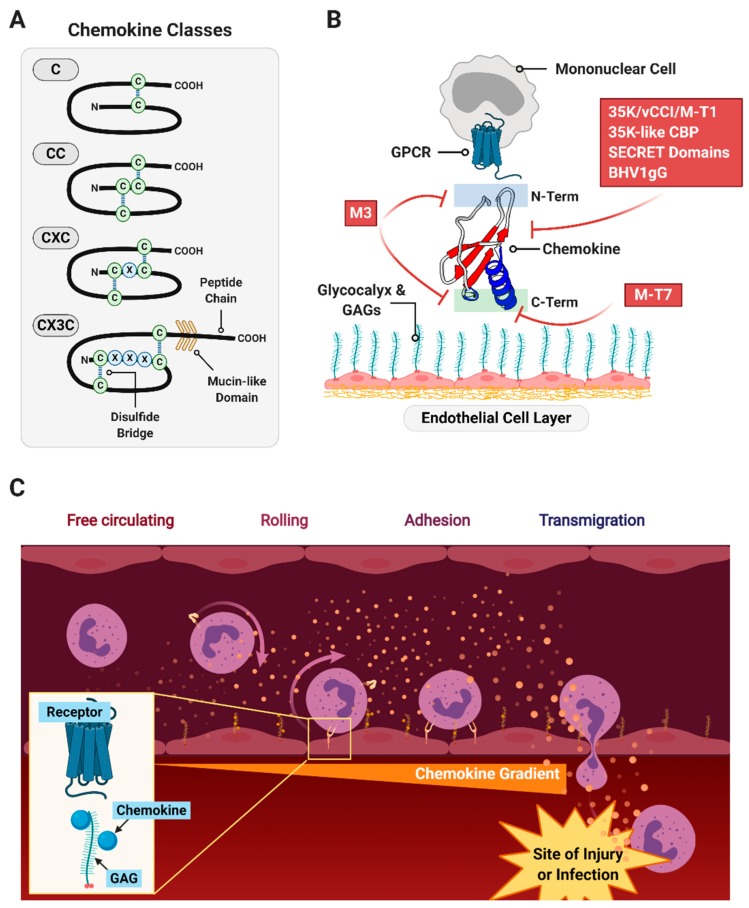
An overview of chemokine function and role. (**A**) Chemokines exhibit highly conserved overall structure with classification by the arrangement of their N-terminal cysteine residues. (**B**) Chemokines bind G protein-coupled receptors (GPCR)-type receptors on chemotactic cells such as mononuclear cells via their N-terminus, and are anchored to tissue matrices and endothelial layers by glycosaminoglycans in the glycocalyx. Viral chemokine signaling modulators are indicated in red boxes with an approximate location of interference indicated. (**C**) Gradients formed by the anchoring of chemokines to the glycocalyx direct circulating immune cells to infiltrate into the site of injury or infection.

Chemokines are small, 8–10 kDa cytokines that direct cell migration, guiding immune system cells to sites of tissue damage from trauma and infection or to areas where foreign tissues have been implanted as in transplants [77,78,79]. Chemokines also direct cell migration in angiogenesis, lymphatic development, wound healing, nerve regeneration and cancer metastases [80]. Chemokines bind glycosaminoglycans (GAGs) and form gradients in the connective tissue and the polysaccharide-containing glycocalyx that surrounds cells in vessels (arteries and veins) and organs. They simultaneously bind 7-transmembrane G protein-coupled receptors (7TMGPCRs, or GPCRs) found on target cells like white blood cells (WBCs) (Figure 4). 

Based on the N-terminal cysteine (C) residues, chemokines are divided into four groups; α (CXC), β (CC), γ (C) and δ (CX3C) chemokines, with X representing intervening amino acids between CC residue groupings (Figure 4A) [81]. In the immune cell response, CXC or α chemokines are generally considered neutrophil or lymphocyte recruiting cytokines, while CC or β chemokines are reported to attract monocytes and T cells [82]. C or γ chemokines (lymphotactins) and δ chemokine (fractalkine) are considered chemoattractant for T cells, NK cells and dendritic cells. However, chemokines are notoriously promiscuous, often crossing over to act on diverging pathways and receptors [80]. 

Chemokine expression is detected at sites of tissue trauma and infection, or in the case of transplants at sites of immune rejection, directing both the necessary early inflammatory (innate immune) and antibody-mediated (immune) cell migration acting both in healing responses and with excess damaging inflammatory and immune responses [83]. Chemokines thus can be upregulated in the ongoing, dysregulated inflammatory and immune responses in diseases such as non-healing skin wounds in diabetics, dermatomyositis, neurotrauma, arthritis, vasculitis and transplant rejection. Increased levels of individual chemokines and chemokine receptors are closely linked with many inflammatory diseases. 

Chemokines bind to GAGs to form a concentration gradient that then directs inflammatory (and in some cases stem) cell binding, leading cells to sites of damage of infection (Figure 4B,C) [84]. The chemokine then binds to GPCR on migrating cells, guiding cells into sites of tissue injury. Chemokines can bind to cellular GPCRs and with GAGs through independent or overlapping binding sites. Chemokines can thus direct immune cells responsible for activating inflammation and immune response or suppressing excess immune responses or inflammation.

There are approximately 50 chemokines and 20 chemokine receptors reported, with the chemokines exhibiting a remarkably conserved three-dimensional fold [79,85]. Chemokine receptor interactions are promiscuous, with most receptors interacting with more than one chemokine and most chemokines more than one receptor [80]. These chemokine–receptor interactions are further refined by binding to specific GAGs, the most prevalent GAGs in the glycocalyx being heparan and chondroitin sulfate.

In a similar fashion as used by viruses for their adoption of serpins, viruses have developed multiple chemokine-targeting strategies including chemokine mimics, chemokine receptor mimics and chemokine binding proteins (CBPs). Two classes of large DNA viruses, poxviruses and herpesviruses, have independently developed highly effective mechanisms that moderate chemokine activity as methods to block host immune responses against viral infection. The viral chemokine receptors mimic host structure; in contrast, the chemokine binding proteins have no sequence homology to native mammalian chemokines or their receptors [86,87,88]. A variety of CBPs are encoded by poxviruses [89,90] and by all three subfamilies (alpha, beta, and gamma) of herpesviruses [91]. Interestingly, the poxvirus-encoded CBPs have a common protein folding not found in mammalian proteins, named the poxvirus immune evasion (PIE) domain, that is also found in poxvirus proteins that bind granulocyte-macrophage colony-stimulating factor, IL-2 or Major Histocompatibility Complex class I molecules [92]. The structure of the gamma herpesvirus M3 protein also displays a novel structure capable of binding chemokines with high affinity [93]. These unique and highly potent virus-derived chemokine modulating proteins further demonstrate the central role of chemokines in immune response, not only for viruses but also in many other invasive organisms including ticks and helminths such as *Schistosoma mansoni*, that also produce CBPs [94,95]. This broad range of pathogen-produced CBPs again demonstrates the benefit of chemokine blockade by pathogens for evasion of host immune cell attack. The CBPs have been extensively tested as potential treatments for immune disorders whereas the receptor mimics have had less attention. We will focus, therefore, on the studies performed to date to investigate poxvirus and herpesvirus derived CBPs as therapeutics. 

Some poxviral and herpesviral CBPs have demonstrated impressive efficacy in preclinical studies. M-T1 and M-T7 are Myxomavirus CBPs; M-T1 interferes with CC chemokine binding to cellular receptors [89] while M-T7 blocks binding of a wide range of C, CC and CXC chemokines to glycosaminoglycans (GAGs) [90]. The deletion of the M-T7 gene in Myxomvirus leads to markedly reduced disease severity in European rabbits [90]. The mouse gammaherpesvirus-68 (MHV-68) encodes the M3 CBP that has the combined function of inhibiting the binding of chemokines to both GAGs and their respective GPCRs [91,96]. In the following section, we review the chemokine modulating protein strategies that have demonstrated preclinical efficacy from both poxviruses and herpesviruses (Table 2).

### 3.1. Poxviral Chemokine Modulators

#### 3.1.1. 35K

The 35K proteins are CBPs found in many poxvirus strains from Vaccinia, Ectromelia, Cowpox and Myxomavirus (where it has been named M-T1) which have a high affinity for CC-class chemokines [117]. When deleted, the absence of the 35K protein causes an increased inflammatory response to viral infection but has minimal effect on overall viral survival [118]. The 35K proteins have been tested and have shown some benefit in atherosclerosis and arthritis models [97,98]. Treatment with M-T1 proved beneficial in reducing pathology in rodent models of angioplasty injury and aortic allograft inflammation [99].

Monkeypox virus (MPV) is an orthopoxvirus with significant homology to variola major [119]. MPV encodes a highly abundant secreted chemokine binding protein referred to as the MPV viral chemokine inhibitor (vCCI), an ortholog of VACV 35K [100]. The canonical target for MPV vCCI is MIP-1α (otherwise known as CCL4). Mice given a high dose of MPV vCCI by intraperitoneal injection were therapeutically protected from experimental allergic encephalomyelitis (EAE) induced by subcutaneous injection of myelin proteolipid peptide (PLP). Interestingly, while the initial phase of the model was blunted versus control-treated animals, mice given MPV vCCI were completely protected from the relapsing phase of the disease [100].

#### 3.1.2. Orf Virus 35K-Like Chemokine Binding Protein

Homologs of the 35K poxvirus proteins have also been isolated in parapoxviruses. This genus of viruses infect primarily ungulates [120], but are also zoonotic, infecting human–animal handlers. The type species, Orf virus (ORFV), was found to secrete two proteins with limited homology with the orthopoxvirus 35K proteins; a granulocyte macrophage-colony stimulating factor and interleukin-2 inhibitory factor (GIF) and a chemokine binding protein (CBP) [121,122]. 

The ORFV GIF exhibits pronounced species specificity, interacting with only ovine and bovine cytokines [121]. This viral protein was shown to inhibit the hematopoietic activity of ovine GM-CSF and T cell proliferation induced by ovine IL-2 [121]. The role of GIF in ORFV infection is not known, but studies on the localized immune response at the site of infection suggest a role in subverting anti-viral immune responses [123]. The GIF gene sequence is present in certain parapoxviruses [124], but functional analysis has shown that the GIF from Pseudocowpoxvirus (PCPV) binds bovine IL-2 and bovine GM-CSF, while the homolog from Bovine papular stomatitis virus (BPSV) does not. 

The ORFV CBP binds with high affinity to human and mouse chemokines from the CC class, and also to selected C and CXC class members [122,125]. Structural studies have revealed that ORFV CBP is dimeric in solution, unusual for a CBP, and has revealed structure determination of complexes with three different cognate binding chemokines (CCL2, CCL3 and CCL7) [125]. Recombinant ORFV CBP, produced from HEK293s, was shown to inhibit monocyte and dendritic cell recruitment *in vitro*, and from the blood to inflamed skin in a murine model, and also reduce T cell activation within the lymphatics [126,127]. Not surprisingly, the deletion of this immune-modulator from the ORFV genome severely attenuates infection of sheep, reducing pustule formation and increasing dermal monocyte and dendritic cell recruitment to the lesion, relative to the wildtype virus [128]. The ORFV CBP is conserved across parapoxviruses, but with low identity across species [99,129,130,131,132]. The CBP encoded by BPSV shows the greatest similarity to that of ORFV, with the recombinant protein binding strongly to chemokines within the CXC, CC and C subfamilies, and inhibiting neutrophil and monocyte recruitment *in vitro* and a murine model of skin inflammation [99]. Intriguingly, three variants have been identified in the genome of Parapoxvirus of red deer of New Zealand (PVNZ) [132], and these CBPs exhibit differences in binding specificity. While the PVNZ CBP112.0 interacted with chemokines from the CXC, CC and C classes of chemokines with nanomolar affinities, CBP112.3 showed a preference for CXC chemokines, and CBP112.6 showed picomolar affinity binding for CC chemokines [132]. In vitro, the recombinant PVNZ CBPs also showed inhibition of neutrophil and monocyte chemotaxis. Structural modeling explained these differences, showing that while the PVNZ CBPs retain the core CBP structure, they differ in their chemokine binding domain [132].

The broad spectrum of activity of the parapoxvirus CBPs has led to investigations as to their therapeutic utility in preclinical models of acute inflammation. Intradermal injection of the BPSV CBP into injured murine skin delayed the influx of neutrophils and reduced the accumulation of MHC-II+ immune cells within the wound bed [99]. Treatment with the BPSV CBP also suppressed inflammation post cerebrovascular accident (stroke) in mice [101]. Decreases were observed in the stroke-induced CCL2 and CXCL2 levels and invasive leukocytes in treated mice relative to controls, along with smaller infarct size and a reduced neurological deficit. The parapoxvirus CBPs with more selective chemokine binding profiles have yet to be tested in preclinical models, but may too offer clinical benefit.

#### 3.1.3. M-T7

M-T7 is a rabbit species-selective interferon-gamma receptor (IFNγR) homolog with specific binding for rabbit IFNγ. M-T7 also binds and inhibits chemokine binding to GAGs for a wide range of mouse and human C, CC and CXC chemokines [90]. When the M-T7 gene is deleted the Myxomavirus becomes a benign self-limiting infection in European rabbits [133]. M-T7 has demonstrated remarkable benefits in vascular injury models from balloon angioplasty to aortic and renal allograft transplants [102,103,104,105,106]. M-T7 treatment given together with cyclosporine reduced transplant rejection, vasculopathy and scarring in rats [104]. When given alone with no additional immunosuppressants, M-T7 reduced aortic allograft inflammation and intimal hyperplasia [105]. In a mouse renal allograft model, M-T7 given alone significantly reduced rejection on analysis by pathologists blinded to treatment and also improved survival, even when given without supplemental immunosuppressant treatments [105,106]. Interestingly, M-T7 activity was lost in the renal allograft model in N-deacetylase sulfotransferase-1 (*Ndst1*) deficient mice. Ndst1 selectively transfers sulfur groups to heparan sulfate GAGs. Of interest, the transplant of donor renal grafts from Ndst1-deficient mice without additional treatment also had markedly reduced rejection providing evidence for new potential immune response pathways and new therapeutic targets for improving diseases with dysregulated inflammation. Treatment with a mutant M-T7, E209I, recovered efficacy in Ndst1-deficient grafts into BALB/c mice, where wildtype was no longer functional. This finding suggests that in scenarios wherein a virus-derived immune modulator has limited efficacy, targeted mutation of susceptible residues may recover function and presents an example for other immune modulators.

#### 3.1.4. Poxvirus SECRET Domains

CrmB and CrmD found in variola virus (VARV) and ectromelia virus (ECTV) respectively are bi-domain proteins that consist of a chemokine binding moiety called SECRET (smallpox virus-encoded chemokine receptor) coupled with a viral tumor necrosis factor receptor (vTNFR). The SECRET domains were initially discovered within the cytokine response modifier (Crm) B and D genes [134]. Other SECRET domains containing proteins (SCPs 1, 2 and 3) have also been identified in CPV (V218, V014 and V201) and ECTV (E12 and E184). The SECRET domains and SCPs have narrow chemokine binding properties inhibiting chemokine receptor binding. CrmD expression specifically in intestinal epithelial cells reduces inflammatory cell migration in vitro and decreased gut inflammation in a transgenic model of Crohn’s like inflammatory bowel disease [107]. In recent work, the SECRET domain from ECTV was fused to the clinically used human TNF receptor Ig Fc fusion protein (Etanercept) and demonstrated its efficacy in a mouse model of arthritis, and possible improvement under certain conditions [108]. This recent work demonstrates that functional domains of virus-derived immune modulators can be exploited in conjunction with other therapeutic proteins to produce synergistic treatments.

### 3.2. Herpesviral CBPs

#### 3.2.1. M3

M3 derived from the mouse gamma herpesvirus-68 (MHV-68) binds to both the GAG-binding and the receptor-binding domains of all classes of chemokines, C, CC, CXC and CX3C [91,96]. M3 N-terminal sequences have partial sequence homology with SECRET domains. M3 protein infusion reduces inflammation when given systemically after aortic allograft transplants in rodent models [102]. Replication-deficient Adenoviral vector expression of M3 administered 5 days after induction of experimental autoimmune encephalitis (EAE) in mice also improved clinical outcomes and reduced evidence of histopathologic changes [109]. M3 has also been reported to increase resistance to diabetes development by inhibiting the priming of diabetogenic cells in the pancreatic lymph nodes and their recruitment into pancreatic islets in transgenic NOD mice, highlighting a key role for chemokines in the development of diabetes [110]. Transgenic expression of M3 attenuated inflammation and suppressed inflammatory infiltration of neutrophils, macrophages and eosinophils in a dextran sulfate-induced colitis model in C57BL6/J background mice [111]. Conditional transgenic mice expressing M3 under the control of doxycycline were resistant to intimal hyperplasia in a model of femoral artery injury [112]. Consistent with this, the expression of M3 from an adenovirus vector has been shown to limit atherosclerosis in apolipoprotein E^−/−^ mice [114]. Adenovirus vectors expressing M2 and M3 gene products have been developed as a method to vaccinate against latent gamma herpesvirus infections [113], uniquely linking a viral chemokine-modulating protein to a vaccine strategy designed to reduce herpes viral latency. Recently, M3 expression has been shown to attenuate inflammation-driven angiogenesis [115] and could represent the basis for the design of novel anti-angiogenic therapies. The manipulation of amino acids near the chemokine binding site by site-directed mutagenesis may be a feasible strategy to modulate the affinity of M3 for particular chemokines that play a more prominent role in particular diseases [135].

#### 3.2.2. Bovine Herpesvirus 1 Glycoprotein G (BHV1gG)

BHV1gG binds to a wide spectrum of murine and human chemokines. When given via adenovirus expression BHV1gG significantly inhibited thioglycollate-induced neutrophil migration into the peritoneal cavity of BALB/c mice and reduced clinical severity and articular damage in K/BxN serum transfer induced arthritis [116]. In contrast, BHV1gG-Ig fusion protein alone did not prevent monocyte invasion into the peritoneum and did not prevent renal monocyte infiltration or nephritis in lupus-prone NZB/W mice, nor did it improve survival after induction of lupus with Ad-IFNα. Importantly, loss of efficacy in this study was found to be associated with the production of neutralizing antibody response [116]. A recombinant version of a similar protein (gG) from herpes simplex virus has been used as an adjuvant to improve the protective immune response to *Helicobacter pylori* vaccination [136].

## 4. Virus-Derived Cytokine and Growth Factor Mimics and Inhibitors

In addition to the binding proteins described above, viruses have developed IFN, cytokine and growth factor mimics and/or inhibitors that can divert host immune responses away from the infecting viral pathogen. Virus-encoded homologs and/or inhibitors of host proteins mimic or target IFNα/β/γ, interleukin 1-beta (IL-1β), interleukin 10 (vIL-10), interleukin 18 (IL-18), complement components and tumor necrosis factor (TNF) and their receptors [119,137]. 

IFN is part of early responses evolved to control viral infection. Several IFN receptor mimics have been discovered that block host IFN activation after viral infections. The Myxomavirus chemokine modulator, M-T7, shares homology with the rabbit IFNγ receptor of selected rabbit species and functionally inhibits IFNγ of rabbits only, but the CBP functions for M-T7 are not limited to rabbits [94]. Viruses also express TNF and interleukin 10 (IL-10) mimics. TNF is considered part of a pro-inflammatory response and is a mediator of immune cell killing, whereas IL-10 is a cytokine often closely associated with reduced inflammation [119,137]. Growth factors drive cell proliferation, angiogenesis and tissue regeneration and a viral vascular endothelial growth factor (vVEGF), was one of the earliest identified “virokines” expressed to modulate host responses by poxviruses [138,139]. Several of these viral mimics and inhibitors, specifically vIL-10, vVEGF and TNF inhibitors have now been successfully assessed as potential therapeutic biologics with marked efficacy in preclinical models (Table 3). 

### 4.1. vIL-10 

A homolog of IL-10 has been identified in ORFV [152], other parapoxviruses [129,130,131] and these vIL-10s share remarkable similarity to mammalian IL-10. Functional characterization of ORFV IL-10, produced as a recombinant protein in HEK-293s, suggests it shares the activities of mammalian IL-10, in contrast to EBV vIL-10 that displays only a subset of activities of human IL-10. This viral protein has been shown to stimulate murine thymocyte and mast cell proliferation [152,153]. ORFV IL-10 also exhibits potent immunosuppressive activity, suppressing murine dendritic cell maturation and T cell activation [127], as well as pro-inflammatory cytokine production in human monocytes [154], murine macrophages [153], equine fibroblasts [155], ovine macrophages, keratinocytes and peripheral blood lymphocytes [156]. The deletion of this gene severely attenuates ORFV, with smaller less severe lesions produced during infection and reinfection of sheep [157].

Mammalian IL-10 is a known regulator of wound healing processes, contributing to scar-free healing [158]. Consistent with this, a phase II randomized clinical trial has shown that intradermal administration of recombinant human IL-10 to human incisional wounds improved the macroscopic appearance of scars [159]. ORFV produces proliferative skin lesions, that even when persistent, resolve with minimal scarring [160]. This observation, and its specific activities, led to ORFV IL-10 being investigating as an anti-scarring therapy in pre-clinical skin wound models. Subcutaneous injection of ORFV IL-10 as with mammalian IL-10 limited scarring of skin wounds in mice, by suppressing pro-inflammatory cytokine production, macrophage infiltration and myofibroblast differentiation [140]. Wound inflammation was also suppressed in experimentally-induced slow-to-heal wounds in horses following subcutaneous delivery of ORFV IL-10 [141], but not after topical hydrogel delivery to fibrotic equine wounds [142]. This vIL-10 may, therefore, provide clinical benefit when applied to human scars, but its route of administration may be critical.

### 4.2. vVEGF

Vascular endothelial growth factors (VEGFs) control blood vessel formation and are critical for development and tissue repair [161]. The mammalian VEGF family comprises of five ligands that each interacts with one or more high-affinity VEGF receptors (VEGFRs). Ligands that activate VEGFR-2 promote blood vessel formation, while those that engage VEGFR-1 to induce inflammation. Consequently, aberrant expression of mammalian VEGFs, or their receptors, has been implicated in numerous inflammatory and vascular diseases, including non-healing wounds, retinopathy and many cancers [162].

The first VEGF homolog discovered in a mammalian virus is encoded by the parapoxvirus, ORFV [163]. Variants of this vVEGF were then identified in all known parapoxvirus species [129,130,164,165,166,167]. The highly proliferative and edematous nature of parapoxvirus skin lesions has been attributed to the expression of the vVEGF. In the absence of a functional vVEGF, ORFV lesions in sheep lack the vascular and epidermal hyperplasia associated with wild-type infections [168,169]. It has thus been hypothesized that the vVEGF supports parapoxvirus replication, by increasing the number of proliferating epidermal cells that can be infected, as well as the supply of nutrients from the vasculature. The vVEGF also likely contributes to environmental spread, as scab formation was reduced in lesions induced by the virus lacking this gene [168]. 

The vVEGFs show extensive genetic diversity from, but the high structural similarity to, the mammalian VEGFs [161,170]. Although the crystal structure of the ORFV protein shows retention of the characteristic homodimeric cysteine knot motif [171], conformational differences are evident in loop regions associated with VEGFR binding, and within the glycosylated C-terminus [165,170,172]. These structural differences explain the unique receptor selectivity of the vVEGFs. Unlike mammalian VEGFs, vVEGFs act primarily through VEGFR2, with limited binding to the other VEGFRs [165,173,174]. The consequences of this receptor-selectivity was seen in *in vitro* assays where the recombinant ORFV vVEGF, produced in *Pichia pastoris* or HEK-293 cells, induced endothelial cell signaling, proliferation and tube formation, but failed to incite monocyte chemotaxis [165,173,175]. *In vivo*, the transgenic overexpression of the ORFV vVEGF in murine skin also increased blood vessel formation, but with limited monocyte infiltration and edema relative to mammalian VEGFs [176].

As the receptor-selective vVEGF retains the reparative, but not inflammatory, properties of mammalian VEGFs, many studies have explored the therapeutic utility of the ORFV vVEGF in preclinical models of tissue damage. Transgenic delivery of the vVEGF to the ischemic myocardium of mice increased ventricular perfusion, without the inflammatory damage associated with mammalian VEGF administration [143]. Subcutaneous injection of the vVEGF as with mammalian VEGF accelerated closure of murine skin wounds, through the enhanced blood vessel and epidermal regeneration [144,145]. Wounds treated with the vVEGF also exhibited reduced inflammation and scarring, which was attributed to an increase in anti-inflammatory IL-10 production [146]. Subcutaneous and topical hydrogel delivery of the vVEGF also increased epithelialization, blood vessel functionality and lesion resolution in experimentally-induced slow-to-heal and fibrotic wounds in horses, when applied in combination with ORFV IL-10 [141,142]. 

Virus derived growth factors have thus evolved such that they exhibit distinct therapeutic advantages over their mammalian counterparts, and have proven effective in preclinical models. A phase 1 clinical trial of recombinant human VEGF (Telbermin) in patients with chronic neuropathic diabetic foot ulcers showed positive trends towards accelerated complete ulcer healing [177], but excess is associated with scar formation [178]. The vVEGF may, therefore, provide the same reparative functions, but with reduced inflammatory complications, thus offering greater clinical benefit.

### 4.3. Viral Tumor Necrosis Factor Inhibitors 

As noted above, the poxviruses express several TNF receptor homologs, specifically cytokine response modifiers, (Crm) B, C, D and E. We have already discussed the C-terminus of CrmB and CrmD, which contain a SECRET chemokine binding domain. These Crm proteins have been examined as potential treatments in full form and recently with the SECRET domain only. Similarly, the TNF-binding domain of CrmB from the variola virus has been shown to protect against collagen-induced arthritis in Wistar rats when delivered as a gene therapy by direct injection of pcDNA plasmid [147]. 

Myxomavirus expresses a TNF binding protein, termed M-T2. In unpublished *in vivo* studies, M-T2 exhibited no therapeutic efficacy in rodent models of angioplasty injury. The TNF inhibitory activity of M-T2 is rabbit species-specific, likely resulting in the inactivity in rodent models of vascular inflammation after angioplasty injury (unpublished observations). Thus, the full characterization of a putative virus-derived immune modulator is an essential component of preclinical validation.

The characterization of virus-encoded TNF receptors may also inspire the construction of modified human TNF receptors used in the clinic. An examination of ligand binding specificities of the TNF binding domain of CrmD, combined with structural data, identified a specific Glu-Phe-Glu motif in the 90s loop in the CrmD cysteine-rich domain 3 responsible for the lack of human LT binding by CrmD [179]. Unlike CrmD, etanercept binds both human TNF and LT, and transfer of the Glu-Phe-Glu motif to etanercept generated a new human TNF inhibitor with reduced anti-human LT activity [180]. This etanercept variant may cause fewer adverse effects compared to the one used in the clinic.

### 4.4. vMIP-II 

Macrophage inflammatory protein-1α (MIP-1α) is a potent, inflammatory chemoattractant. Viral adoption of this chemokine (vMIP-II) can be found in numerous human herpesviruses, resulting in antagonism of mammalian MIP-1α signaling. Initial studies with vMIP-II found that it competed for binding with MIP-1α at the CCR1 and CCR5 receptors, as well as with MCP-1 (CCL2) at the CCR2 receptor [181]. In the same work, it was found that vMIP-II competed with SDF-1 (CXCL12) at the CXCR4 receptor and had a weak activity to bind CXCR2. Thus, in addition to the antagonism of the MIP-1α pathway, vMIP-II has broader effects on a wider range of chemokine signaling. The therapeutic translation of vMIP-II has been tested in numerous preclinical models. Intracerebroventricular injection of vMIP-II reduced infarct volume dose-dependently at 48 h after a 1-h middle cerebral artery occlusion followed by a reperfusion period [148]. In contrast, injection of the mammalian analog, MIP-1α, increased infarct volume in the same study. Thus, the putative expanded function of the viral version of the same chemokine conferred potential for additional therapeutic function. In another study, two daily intravenous injections of vMIP-II protected rats in a model of anti-glomerular basement membrane-induced glomerulonephritis, with a reduction of activated leukocytes and attenuated proteinuria [149]. Continuous infusion of vMIP-II decreased infiltrating hematogenous cells at the site of injury in a spinal cord contusion injury in rats, with associated reductions in neuronal loss and gliosis [150]. However, this study did not include an assessment of locomotor function, thus it remains to be seen whether chemokine antagonism with vMIP-II improves functional recovery, as seen with the poxvirus chemokine modulator M-T7 [61]. Gene transfer of vMIP-II by direct injection of plasmid DNA improved cardiac allograft survival when hearts were placed in the subcutaneous position of the ear pinnae of CBA/J recipients [151].

### 4.5. MC148

MC148 is a CC-class chemokine expressed by the poxvirus *Molluscum contagiosum*, with an affinity for both CC and CXC chemokine receptors [182]. In the same study mentioned above with vMIP-II, direct injection of MC148 in cardiac allografts significantly improved survival from a mean of 13.4 days to a mean of 25.5 days [151].

## 5. Other Inhibitors

### 5.1. Viral Complement Control Proteins

Complement proteins are serine proteases that drive cell killing through activation of a membrane attack complex in normal, innate and acquired immune cell responses. Complement proteases interact with receptors C3Ra and C5aR, so-termed anaphylatoxin receptors, to form a membrane attack complex that targets cells and causes cell lysis. The same complement attack complex C5b9 is implicated in neuronal damage [183]. The membrane attack complex leads to inflammatory cell chemotaxis, opsonization and cell lysis. 

The vaccinia virus complement control protein, VCP, first identified by Kotwal and Moss, is an inhibitor of the classic and alternative complement pathways with structural similarity to C4b and functional similarity to CR1 [184]. It is also reported to bind heparan sulfate GAGs [185]. Treatment with VCP improved sensorimotor control in rodent models of severe brain trauma but did not improve cognitive function [84]. VCP treatment also improved hyperacute rejection in guinea pig to rat heart xenotransplantation models [186]. Suggestive work in a recent study found that direct injection of VCP to the rat retina might reduce photoreceptor cell death by complement inhibition after photo-oxidative retinal degeneration and explored delivery via a slow-release substrate [187]. 

### 5.2. M013L

The inflammasome is a central pathway in the innate immune response to pathogens and damage. Activation of the inflammasome via a prion-like mechanism involving the assembly of a central sensor protein (such as a NOD-like receptor), an adapter protein called Apoptosis-associated Speck-like protein containing a CARD domain (ASC) and an inflammatory caspase such as caspase-1, results in the processing and release of inflammatory cytokines IL-1b and IL-18 and an inflammatory cell death called pyroptosis [188,189]. Myxomavirus-expressed M013L binds and inhibits both NFκB in the inflammatory response as well as the pyrin domain of ASC in the inflammasome pathway [190]. M013L fused to a cell-penetrating TAT domain has proven effective when expressed via AAV vector for reducing endotoxic uveitis in mouse eye inflammation models [191]. In related work, TAT-M013 was effective against experimental autoimmune uveoretinitis in mice induced by immunization with interphotoreceptor retinoid-binding protein residues 161–180 in Freund’s adjuvant [17].

### 5.3. VACV TLR Signaling Inhibitor-Derived Peptides

Vaccinia virus encodes numerous inhibitors of TLR signaling, including A46 and A52. A multi-peptide screen found that one peptide from A46, named VIPER (viral inhibitor peptide of TLR4), was a potent inhibitor of TLR4-mediated responses via specific binding to the proteins TRIF-related adaptor molecule (TRAM) and MyD88 adaptor-like (Mal) [192]. VIPER dose-dependently inhibited LPS-induced IL-12/23 p40 secretion in BALB/c mice, suggesting a therapeutic potential for peritonitis-like diseases. In another study, the P13 peptide from VACV A52 therapeutically inhibited heat-inactivated *Streptococcus pneumoniae*-induced middle ear inflammation (otitis media) in BALB/c mice [193].

## 6. Future Directions

These virus-derived immune-modulating biologics represent a new therapeutic direction. However, of the wide range of virus-derived immune-modulating proteins, only a few have been explored as potential therapeutics. While this review has focused primarily on poxvirus and herpesvirus derived agents, immune-modulating factors from other viruses are only just beginning to be identified and tested in preclinical studies. For example, a synthetic peptide derived from the murine leukemia virus (MLV) retrovirus is reported to suppress inflammatory damage in two models of skin inflammation in mice [194]. Other organisms including other viruses, fungi and parasites such as malaria and insects have proven immune-modulating actions that have not yet been tapped as potential sources for therapeutics. Potent immune modulators have been identified in many organisms. For example, Evasins are chemokine-binding proteins isolated from tick salivary glands [95]. Evasin-3 was effective as a treatment to reduce stroke in a mouse carotid occlusion model [195] and a single administration was found to prevent myocardial reperfusion injury in mice [196]. In the tick pathogen *Haemaphysalis longicornis*, two new serpins Hlserpin-a and HlSerpin-b have been identified as inhibitors of cathepsins B and G, reducing joint inflammation in a mouse model of collagen-induced arthritis [197]. The same study found that RCL peptides derived from the *H. longicornis* serpins were also therapeutically effective, similar to Serp-1 RCL-derived peptides [197]. In plants, the cucumber mosaic virus expresses a movement protein (MP) in *Arabidopsis thaliana* and *Nicotinia benthamiana* that has a unique capacity to inhibit immune responses in plants [198]. MP suppresses reactive oxygen species (ROS) production induced by pathogen-associated molecular patterns (PAMPs), suppressing PAMP responses to bacteria-derived peptides and fungal-derived chitin and may represent yet another branch in the tree of life with an uninvestigated stock of putative therapeutics that could potentially be translated to mammals. 

**Figure 5:**
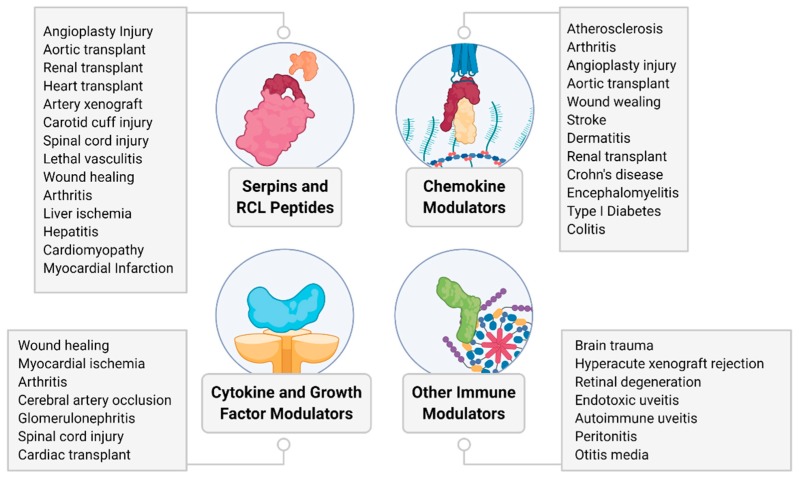
Summary overview of diseases and conditions tested for therapeutic efficacy of virus-derived proteins in preclinical models discussed in this review.

Throughout broad biological systems, pathogens such as viruses have adopted molecules to combat host immunity. Virus-derived immune-modulating biologics provide a new and highly potent class of naturally derived drugs now ready for investigation as treatments for (auto) immune and (auto) inflammatory disorders (Figure 5).

## Figures and Tables

**Table 1:** Viral Serpins and Serpin-derived Peptides with therapeutic efficacy.

Serpin	Virus	Targets	Models	Clinical	Refs.
Serp-1	Myxoma virus	uPA, tPA, FXa, Plasmin, Thrombin (w/ heparin); Requires uPAR function *in vivo*	Aortic balloon angioplasty injury; Aortic transplant; Renal transplant; Heart transplant; Temporal artery xenograft; Carotid cuff compression; Spinal cord injury; MHV-68 lethal vasculitis; Wound healing; Collagen-induced arthritis	Phase I/IIa for Acute coronary syndrome—coronary stent implant	[27,48,52,53,54,55,56,57,58,59,60]
Serp-2	Myxoma virus	Caspase-1, -8, -10, Granzyme B	Aortic transplant; Liver ischemia-reperfusion injury; Carotid cuff compression	N.D.	[50,51,59,61]
CrmA	Cowpox virus	Caspase-1, -8, Granzyme B	Anti-Fas hepatitis; ConA hepatitis; Doxorubicin cardiomyopathy; LAD ligation MI	N.D.	[62,63,64,65]
Serp-1 RCL peptide: S-7	Myxoma virus	Unknown; inhibits PAI-1 and NSP activity	MHV-68 lethal vasculitis; Aortic transplant	N.D.	[66]
Serp-1 RCL peptides: MPS7-8,9	Myxoma virus	Unknown; inhibit Thrombin-ATIII complex formation	MHV-68 lethal vasculitis	N.D.	[33]

N.D., Not done. MPS7, modified peptide S-7.

**Table 2:** Viral chemokine-modulating factors with therapeutic efficacy.

Protein	Virus	Targets/Function	Models	Refs.
35K; vCCI (MPV); M-T1 (MYXV);	Vaccinia virus; Myxoma virus; Monkeypox virus; Ectromelia virus; Cowpox virus	CC chemokines, CCL3/MIP-1α for MPV vCCI; prevents receptor binding	Atherosclerosis; Arthritis; Angioplasty injury; Aortic transplant; Experimental allergic encephalomyelitis	[97,98,99,100]
35K-like CBP	Orf virus; Bovine papular stomatitis virus	C, CC, CXC chemokines; prevents receptor binding	Wound healing; Stroke; Skin inflammation	[99,101]
M-T7	Myxoma virus	C, CC, CXC chemokines; prevents GAG binding	Balloon angioplasty injury; Aortic transplant; Renal transplant	[102,103,104,105,106]
SECRET domains	Variola virus; Ectromelia virus	CC, CXC chemokines; prevents receptor binding	Genetic Crohn’s like disease; Arthritis	[107,108]
M3	Mouse gamma herpesvirus-68	C, CC, CXC, CX3C chemokines; prevents receptor binding	Experimental allergic encephalomyelitis; NOD Type I diabetes; DSS colitis; Arterial injury; Vaccine adjuvancy	[109,110,111,112,113,114,115]
BHV1gG	Bovine herpesvirus-1	CC, CXC chemokines; prevents receptor binding	Serum transfer-induced arthritis	[116]

**Table 3:** Viral cytokine and growth factor mimics and inhibitors with therapeutic efficacy.

Protein	Virus	Target Protein Mimic/Inhibitor	Models	Refs.
vIL-10	Orf virus	IL-10 mimic	Wound healing	[140,141,142]
vVEGF	Orf virus; all parapoxviruses	VEGF mimic	Wound healing; Myocardial ischemia	[143,144,145,146]
CrmB TNF-binding domain	Variola virus; Ectromelia virus; Cowpox virus	TNF inhibitor by receptor decoy	Collagen-induced arthritis	[147]
vMIP-II	Herpesviruses	MIP-1α mimic	Cerebral artery occlusion; Anti-GBM glomerulonephritis; Spinal cord injury; Cardiac transplant	[148,149,150,151]
MC148	Molluscum contagiosum	CC-class chemokine mimic	Cardiac transplant	[151]
